# Icephobic Performance of Combined Fluorine-Containing Composite Layers on Al-Mg-Mn–Si Alloy Surface

**DOI:** 10.3390/polym13213827

**Published:** 2021-11-05

**Authors:** Vladimir S. Egorkin, Dmitry V. Mashtalyar, Andrey S. Gnedenkov, Valeriia S. Filonina, Igor E. Vyaliy, Konstantine V. Nadaraia, Igor M. Imshinetskiy, Evgeny A. Belov, Nikolaj V. Izotov, Sergey L. Sinebryukhov, Sergey V. Gnedenkov

**Affiliations:** Institute of Chemistry, Far Eastern Branch of the Russian Academy of Sciences, 690022 Vladivostok, Russia; madiva@inbox.ru (D.V.M.); asg17@mail.com (A.S.G.); filonina.vs@gmail.com (V.S.F.); igorvyal@gmail.com (I.E.V.); nadaraiakv@mail.ru (K.V.N.); igorimshin@gmail.com (I.M.I.); belov_eal@mail.ru (E.A.B.); nikolaj.izotov@mail.ru (N.V.I.); sls@ich.dvo.ru (S.L.S.); svg21@hotmail.com (S.V.G.)

**Keywords:** aluminum, protective coating, plasma electrolytic oxidation, composite coating, ice adhesion

## Abstract

This paper presents the results of an evaluation of anti-icing properties of samples obtained by plasma electrolytic oxidation (PEO) with a subsequent application of superdispersed polytetrafluoroethylene (SPTFE) and polyvinylidenefluoride (PVDF). A combined treatment of the samples with SPTFE and PVDF is also presented. It is revealed that impregnation of a PEO layer with fluoropolymer materials leads to a significant increase in surface relief uniformity. Combined PVDF–SPFTE layers with a ratio of PVDF to SPTFE of 1:4 reveal the best electrochemical characteristics, hydrophobicity and icephobic properties among all of the studied samples. It is shown that the decrease in corrosion current density *Ic* for PVDF–SPFTE coatings is higher by more than five orders of magnitude in comparison with uncoated aluminum alloy. The contact angle for PVDF–SPFTE coatings attain 160.5°, which allows us to classify the coating as superhydrophobic with promising anti-icing performance. A treatment of a PEO layer with PVDF–SPFTE leads to a decrease in ice adhesion strength by 22.1 times compared to an untreated PEO coating.

## 1. Introduction

Impacting the icephobic properties of a surface is a particular complex of measures directed at its modification to provide the lowest adhesion of water droplets at low temperatures to avoid ice crystal growth. Superhydrophobic surfaces (i.e., surfaces characterized by a high value of the contact angle (CA), a low contact angle hysteresis and a sliding angle of less than 10°) can retain an air layer between water and structural elements, forming the surface relief and preventing droplet retention on the surface of the material [1,2,3,4,5,6,7,8]. It is noted that superhydrophobic surfaces enhance the rebound of falling liquid droplets at low substrate temperatures and high relative humidity [9]. Moreover, such coatings reduce the adhesion strength of the ice to the substrate, delay water freezing, and reduce or even completely prevent the nucleation and accumulation of ice and snow on the treated surfaces [7,10,11,12]. Taking these into account, it can be stated that surface anti-icing properties and hydrophobicity are closely related, and superhydrophobic surfaces exhibit promising anti-icing performance [13]. There are several approaches used to impart a surface’s anti-icing properties on the basis of its non-wettability, including application of hydrophobic materials without surface texturing, nanostructured hydrophilic materials and a combination of these techniques [14,15,16,17]. Such strategies make it possible to achieve the formation of a multimodal surface relief, reduce the surface energy and, consequently, attain a stable superhydrophobic state. Among different methods of forming superhydrophobic surfaces, strongly adherent multifunctional coatings based on plasma electrolytic oxidation treatment are of great potential and simplicity for light metals, such as aluminum and its alloys. The combination of a complex surface topography of the PEO layer and a subsequent post-treatment with various compounds facilitates the formation of a multimodal roughness of surface relief, providing a wetting resistivity [18,19,20,21,22,23]. Polytetrafluoroethylene (PTFE) is a perfluorinated organic compound characterized by very stable chemical properties due to its molecular structure, containing carbon (C) and fluorine (F) (–CF_2_–CF_2_–groups) with the extreme strength of both carbon–carbon (backbone group) and carbon–fluorine (pendant group) bonds. The presence of fluorine on the bond ends provides polytetrafluoroethylene with nonpolarity and both low surface energy and a low friction coefficient [24,25]. The low surface energy of the material and the formation of multimodal roughness on the surface sample can be key to the achievement of a stable superhydrophobic state and anti-icing performance, respectively. There are different studies presented in the literature aimed at a reduction in ice adhesion by modification of different surfaces with PTFE. For example, Wang et al. document PTFE application via the electrostatic attraction self-assembly method to reduce ice adhesion to 6061 aluminum alloy [25]. Eshaghi et al. fabricated ZnO/PTFE-SiO_2_ nano-composite thin films on copper substrates by immersion with a subsequent heat treatment [26]. The PTFE-containing surface layer was obtained by Chao et al. by immersion of titanium substrate with a nanostructured surface, formed by laser treatment, into fluoropolymer solution [27]. However, according to Hejazi et al. [8], the surface is determined as anti-icing when it provides an ice adhesion strength value lower than 100 kPa. Therefore, not all of the PTFE-based coatings presented in the literature can be regarded as prior choice for an anti-icing surface modification. Moreover, due to its insolubility in organic solvents, a successful commercial use of PTFE-containing suspensions to form anti-icing coatings is handicapped, and there is a need for an inter-agent to form the strong matrix for retention of the PTFE component and improve the properties of the formed coatings. A partially fluorinated fluoropolymer polyvinylidene fluoride can be used as an inter-agent, forming the polymeric matrix for a subsequent filling with PTFE due to the presence of hydrogen molecules in its structure (the molecule is formed by –CF_2_–CH_2_–groups) [24]. The addition of PVDF also provides increased mechanical strength compared to a single perfluorinated polymer [28]. It also has higher abrasion resistance as well as resistance to both creep under long-term stress and fatigue during cyclic loading [28], which can provide the durability of anti-icing performance of formed surface layers due to the preservation of the surface relief structure even after mechanical action. There are some examples of using PVDF to form coatings with anti-icing properties. Peng et al. [29] describe the formation of a superhydrophobic coating on a wind turbine blade using the N,N-dimethylformamide-based solution of PVDF and NH_4_HCO_3_. Zhang et al. [30] formed PVDF–PTFE composite membranes via sequential treatment in PVDF and PTFE solutions for further fabrication of slippery liquid-infused porous surfaces.

The current work is aimed at the formation of fluoropolymer-containing, highly adherent composite surface layers with PVDF polymeric matrix and SPTFE filler to impart the anti-icing properties of AMg3 aluminum alloy. Novelty is ensured by the fact that such polymeric compositions with a PEO layer as a matrix and superdispersed polytetrafluoroethylene (spherical particles composed of nanosheets, obtained using gas-dynamic thermal destruction method [31]) dispersed in PVDF as a texturing and modifying agent were formed and investigated for the first time.

## 2. Materials and Methods

### 2.1. Samples

AMg3 aluminum alloy (ALKENNY, St. Petersburg, Russia) rectangular plates sized 50 × 50 × 1.5 (mm^3^) were used as samples for coatings formation. The alloy belongs to Al–Mg–Mn–Si system [32], and its elemental composition is (wt. %): 3.75 Mg, 0.78 Si, 0.38 Mn, to 0.43 Fe, 0.10 Zn, 0.10 Cu, 0.10 Ti, 0.05 Cr, balance—Al. All samples were subsequently ground with SiC sandpapers from 240 grit of the first one to 1200 grit of the last one, washed with distilled water, air dried and degreased with alcohol.

### 2.2. PEO Processing

Plasma electrolytic oxidation was carried out in the electrolyte, containing 20 g/L Na_2_SiO_3_·5H_2_O, 10 g/L Na_2_B_4_O_7_·10H_2_O, 2 g/L NaF, and 2 g/L KOH, dissolved in deionized water. All samples were processed in two stages in bipolar mode, with periodic alternation of cathodic and anodic pulses. At the first stage, the voltage in the anodic period increased from 30 to 540 V with a sweep rate of 3.4 V/s, and then the voltage value was fixed at 540 V for 750 s. In the cathodic period the current was held at 6.5 A for 900 s. The duty cycle was equal to 1. The presented electrolyte composition and mode parameters allowed us to obtain thick surface layers with a developed surface.

### 2.3. Formation of Polymer-Containing Layers

Polyvinylidenefluoride (Merck, Darmstadt, Germany) and superdispersed polytetrafluoroethylene (Vladivostok, Russia, ^®^Forum) were used as fluoropolymer materials for the formation of icephobic composite layers.

The SPTFE layer was applied from a 15 % SPTFE suspension in isopropyl alcohol (Merck, Darmstadt, Germany) using the dip-coating technique. Based on the results of previous studies [33,34,35], the most uniform surface layers were obtained by dipping a sample into a continuously stirred suspension for 10–15 s, followed by drying until solvent evaporation (nearly 20 min at 25 °C in ambient conditions). The samples were then heat treated at 315 °C for 15 min to provide the best pores filling with a fluoropolymer material. The coating (CC-SPTFE) was formed by a threefold deposition to obtain the optimal complex of protective properties [36].

The PVDF layer was formed by dipping the PEO-coated sample in the 6 % PVDF solution in *N*-methyl-2-pyrrolidone (Merck, Darmstadt, Germany) followed by drying at 70 °C for 2 h. PVDF-containing coating was also formed by a threefold application (CC-PVDF).

To form the combined PVDF–SPFTE coatings, SPTFE particles were added in the PVDF solution in different proportions (PVDF:SPTFE)—1: 1, 1: 2, 1: 3, 1: 4, 1: 5. The samples were dipped in the PVDF–SPFTE solutions once and then dried at 65 °C for 3 h.

The applied thermal treatment modes made it possible to level the difference in viscosity of the applied fluoropolymer compositions in order to achieve the least thermal destruction of the formed polymer layer, which, in turn, allowed heat treatment to have the least effect on the properties of PVDF and PVDF–SPFTE coatings.

### 2.4. Surface Characterization

Morphology of the PEO coatings was investigated by scanning electron microscopy (SEM). SEM images of the sample surfaces were obtained using Zeiss EVO 40 scanning electron microscope (Carl Zeiss Group, Oberkochen, Germany) with Silicon Drift Detector X-MaxN 80 (Oxford Instruments NanoAnalysis, Concord, MA, USA). The study of morphology of the coatings was performed in the Far Eastern Center for Electron Microscopy, Federal State Institution of Science A.V. Zhirmunsky National Scientific Center of Marine Biology of the Far Eastern Branch of the Russian Academy of Sciences (Vladivostok, Russia).

### 2.5. Wettability Measurements

A 3 wt.% NaCl solution in distilled water was used as a testing liquid for wettability measurements. The wettability of the obtained coatings was evaluated by sessile drop method on drop shape analyzer DSA100 (KRÜSS, Neuss, Germany). The angle between the drop baseline and the tangent at the three-phase contact point was measured. The software enables CA determination using several methods of the drop shape approximation (ellipse, tangent, circle, Young–Laplace etc.) [37]. With a sessile drop being under the effect of gravity, the curvature of its shape is affected by the hydrostatic pressure resulting from the weight of the liquid. At the same time, the radii of curvature of the surface change as a function of height. In accordance with the recommendations [20,37,38,39], the Young–Laplace method was used to measure the contact angle. This method takes into account the gravitational distortion of liquid drops forming under their own weight. The parameters of an equation system, which models the shape of the sessile drop, are determined by means of numerical analysis. KRÜSS recommends using this method for symmetrical droplets with the contact angle from 10 to 180 degrees and the drop weight from small to very large.

### 2.6. Localized Electrochemical Studies

Localized techniques (scanning vibrating electrode technique (SVET) and scanning ion-selective electrode technique SIET) [40,41] (Applicable Electronics, Sandwich, MA, USA) were used to obtain information about the local corrosion process kinetics on the surfaces of formed protective coatings in 3 wt.% NaCl solution. The exposed surface area of the sample was 1 × 1 (mm^2^).

The SVET probe, located at 100 ± 5 μm above the scanned surface, was an insulated wire made of platinum–iridium alloy with a layer of platinum black deposited on its tip with a diameter of 15 μm. The probe peak-to-peak vibration amplitude was equal to 16 μm (Z-axis) and 17 μm (X-axis) with frequency values of 99 Hz (Z-axis) and 160 Hz (X-axis). The data from vertical (Z-axis) component of vibration were analyzed.

The H^+^-selective microelectrode was used for SIET measurements. It was made from single-barreled glass capillary with an outer diameter of 1.5 mm. The diameter of the opening of the conical tip of glass capillary was 2.0 ± 0.5 μm. The silanized capillaries were backfilled with solution of 0.01 M KH_2_PO_4_ in 0.1 M KCl. Ionophore-based membrane selective for H^+^ (Fluka, Ref. 95293) [42] was embedded in the capillary tip. The external reference electrode was a silver chloride Ag/AgCl/0.1 M KCl, 0.01 M KH_2_PO_4_ electrode. The ion-selective microelectrode was located at 50 ± 5 μm above the studied surface. The microelectrodes were mounted on the SVET/SIET dual-head stage to perform the quasi-simultaneous measurements of local electrochemical processes. The control of SVET/SIET measurements was carried out by means of LV-4 software (Science Wares, Falmouth, MA, USA) [41].

After isolating sample edges with beeswax, the studied areas of specimens were about 6 mm^2^.

### 2.7. Conventional Electrochemical Measurements

Electrochemical properties of coatings were investigated using electrochemical system ModuLab XM ECS (Solartron analytical, Farnborough, UK). Measurements were carried out in a three-electrode cell in a 3 wt.% sodium chloride solution at room temperature (25 ± 1 °C). Platinum mesh was used as a counter electrode, and saturated calomel electrode was used as a reference electrode. The exposed area of samples was 1 cm^2^. The samples were held in a solution for 60 min prior to all electrochemical tests to achieve a steady state. To record the impedance spectrum, a sinusoidal signal with an amplitude of 10 mV (rms) was used. The experiments were carried out in the frequency range from 0.1 MHz to 0.01 Hz at a logarithmic sweep of 10 points per decade. The potentiodynamic polarization measurements were carried out at a scan rate of 1 mV·s^−1^ in the range from *Ec*—0.25 V to *Ec* + 2 V. The Levenberg–Marquardt (LEV) method was used to fit the experimental polarization curves (i.e., values of corrosion potential, *Ec*, and current density, *Ic*) by the following equation:

*I* = *Ic*·(10^(*E*−*Ec*)/*βa*^ + 10^−(*E*−*Ec*)/*βc*^) (1)

which gives best-fit values of corrosion potential, *Ec*, and corrosion current density, *Ic*.

### 2.8. Ice Adhesion Tests

**Figure 1:**
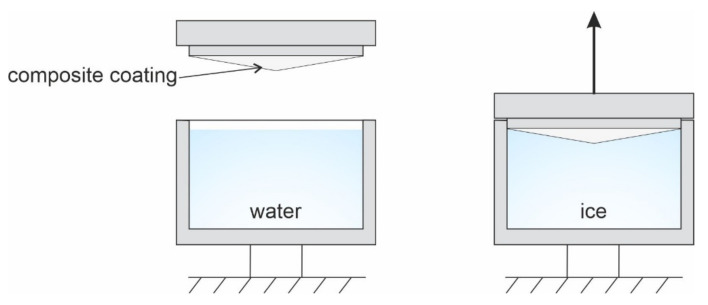
Schematic representation of the experimental setup and the sample used to determine the ice pull adhesion strength.

Ice adhesion to the studied coatings was evaluated by a pull-off test using the samples with a specially developed conical shape (Figure 1), which made it possible to increase the accuracy and repeatability of measurements. The experimental setup models the situation when the rod with the flat tip is immersed into the liquid. The conical shape of the sample with the tip is equal to about 176° due to the necessity to remove the air bubbles inevitably entrapped at the surface. It is assumed that at first estimation, such shape does not lead to a significant influence of the shear adhesion forces’ component in determining the tensile adhesion strength. In addition, this method is reproducible and allows a numerical comparison between different coatings. The samples were oxidized and treated with fluoropolymers. After that, the conical part of the samples was placed in an aluminum cell filled with bidistilled water, and this assembly was then frozen at −20 °C. Pull tests were carried out in a thermostatic chamber at −20 °C. After the test, the samples were heated to room temperature and, without additional drying, they were then subjected to repeated freezing and pull testing. Each sample was subjected to this procedure 10 times.

## 3. Results and Discussion

**Figure 2:**
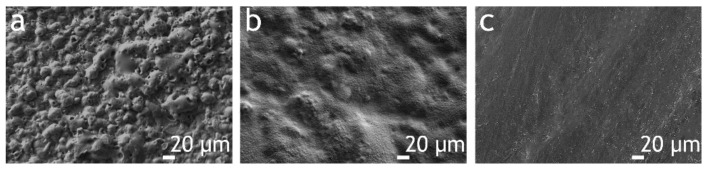
SEM images of a surface with different coating types: (**a**) PEO; (**b**) CC-SPTFE; (**c**) CC-PVDF.

**Figure 3:**
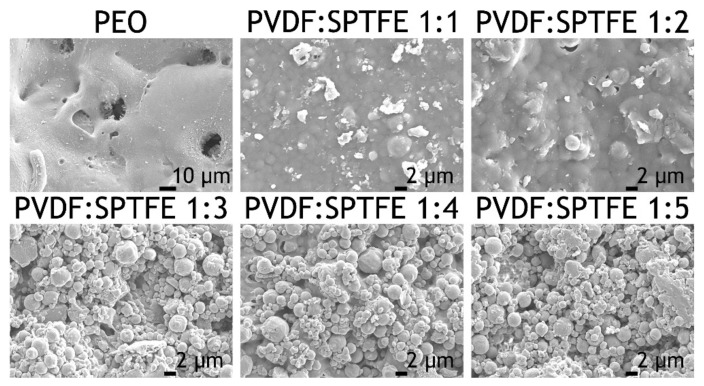
SEM images of the PEO layer and composite coatings formed on the AMg3 aluminum alloy.

Analysis of SEM images of the PEO coating (Figure 2a and Figure 3) indicates that the base PEO layer has a developed surface morphology, expressed in the presence of a large number of pores and microdefects. The application of CC-PVDF and CC-SPTFE layers (Figure 2b,c) leads to a significant increase in surface relief uniformity and a decrease in the number of microdefects and pores. The study of the morphology of combined coatings allows us to conclude that an incorporation of fluoropolymer materials in a ratio of 1:1 does not lead to significant increase in surface uniformity (Figure 3). As the concentration of SPTFE microparticles in a PVDF solution increases, there is an increase in the uniformity of surface morphology and in the number of pores and microdefects filled with fluoropolymers (Figure 3). Moreover, when the proportion of PVDF and SPTFE reaches 1:3 and 1:4, the agglomeration of particles is observed, which leads to the formation of a multimodal surface roughness (Figure 3). However, when the ratio of PVDF to SPTFE reaches 1:5, the microparticles form too-large agglomerates, which probably exclude the formation of a surface relief, allowing us to achieve the stable superhydrophobic state.

Evaluation of the wettability of the formed coatings revealed that the values of the contact angle can be controlled by a change in the concentration of SPTFE microparticles in a PVDF solution (Table 1). Thus, the PEO coating is hydrophilic with a CA of 28.3° ± 1.2°. CC-PVDF is characterized by CA of 100.5° ± 1.8°, which is due to the absence of a multimodal surface roughness (Figure 2c). The addition of SPTFE microparticles in a 1:1 ratio leads to an increase in CA values up to 110.7° ± 0.5°. When the PVDF:SPTFE ratio reaches 1:3, the forming of surface microstructures contributes to an increase in water resistance with CA value of 130.5° ± 2.1°. The highest contact angle 160.5° ± 3.8° was registered for PVDF–SPFTE layers with a 1:4 ratio of PVDF to SPTFE, allowing us to classify this coating as superhydrophobic. The CA value for CC-SPTFE is 155.0° ± 1.5°.

Further results are presented for the following types of coatings: CC-PVDF, CC-SPTFE and coatings obtained with a PVDF–SPTFE ratio of 1:4 as the best among all of the studied ratios (1:1, 1:2, 1:3, 1:4 and 1:5).

**Figure 4:**
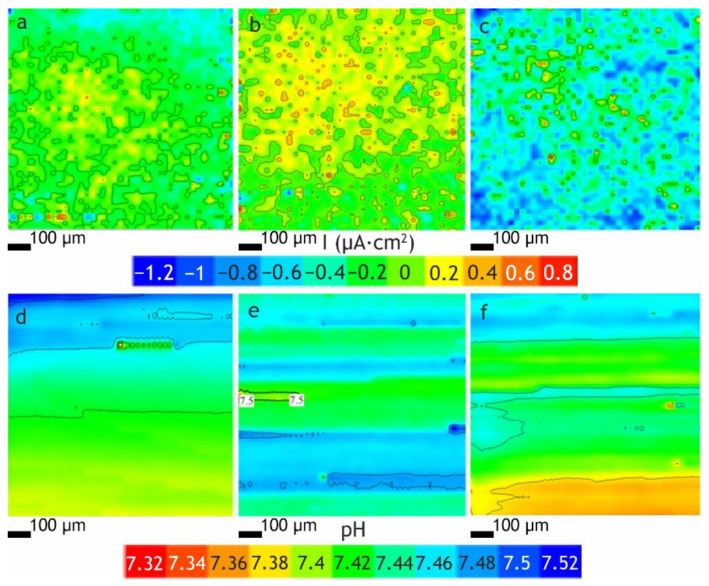
SVET and SIET maps of the distribution of (**a**–**c**) local current densities and (**d**–**f**) pH over the surface of the samples, respectively, with (**a**,**d**) CC-PVDF, (**b**,**e**) CC-SPTFE and (**c**,**f**) coating with PVDF–SPTFE ratio 1:4 after 12 h of scanning.

Using the SVET and SIET methods, the distributions of the local current densities and pH, respectively, over the local areas of the studied samples were established. Analysis of the SVET data (Figure 4a–c) indicates low electrochemical activity of the composite polymer coated samples (after 12 h of scanning, the change in current density of Δ*i*_max_ was 0.4 μA·cm^−2^), which is explained by the absence of local electrochemical processes. Local pH values were in the range of 7.3–7.6, also indicating the absence of intense electrochemical processes associated with sample corrosion. Analysis of the obtained data allowed us to identify the high protective properties of the formed layers.

**Figure 5:**
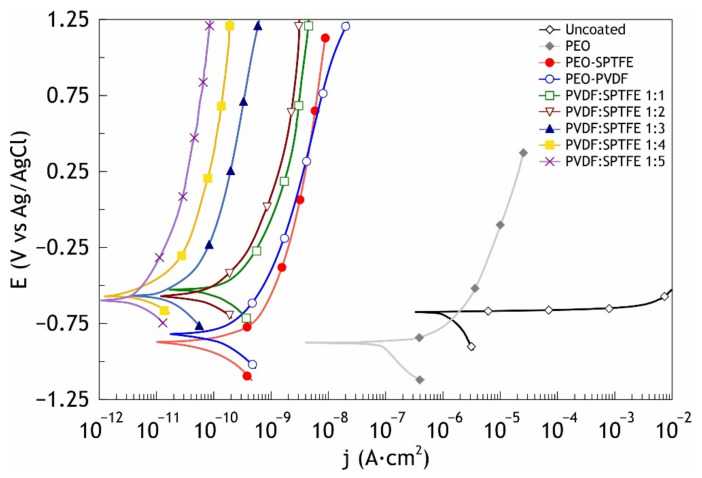
Potentiodynamic polarization curves obtained in 3 wt.% NaCl for the bare AMg3 aluminum alloy and the coated samples.

**Figure 6:**
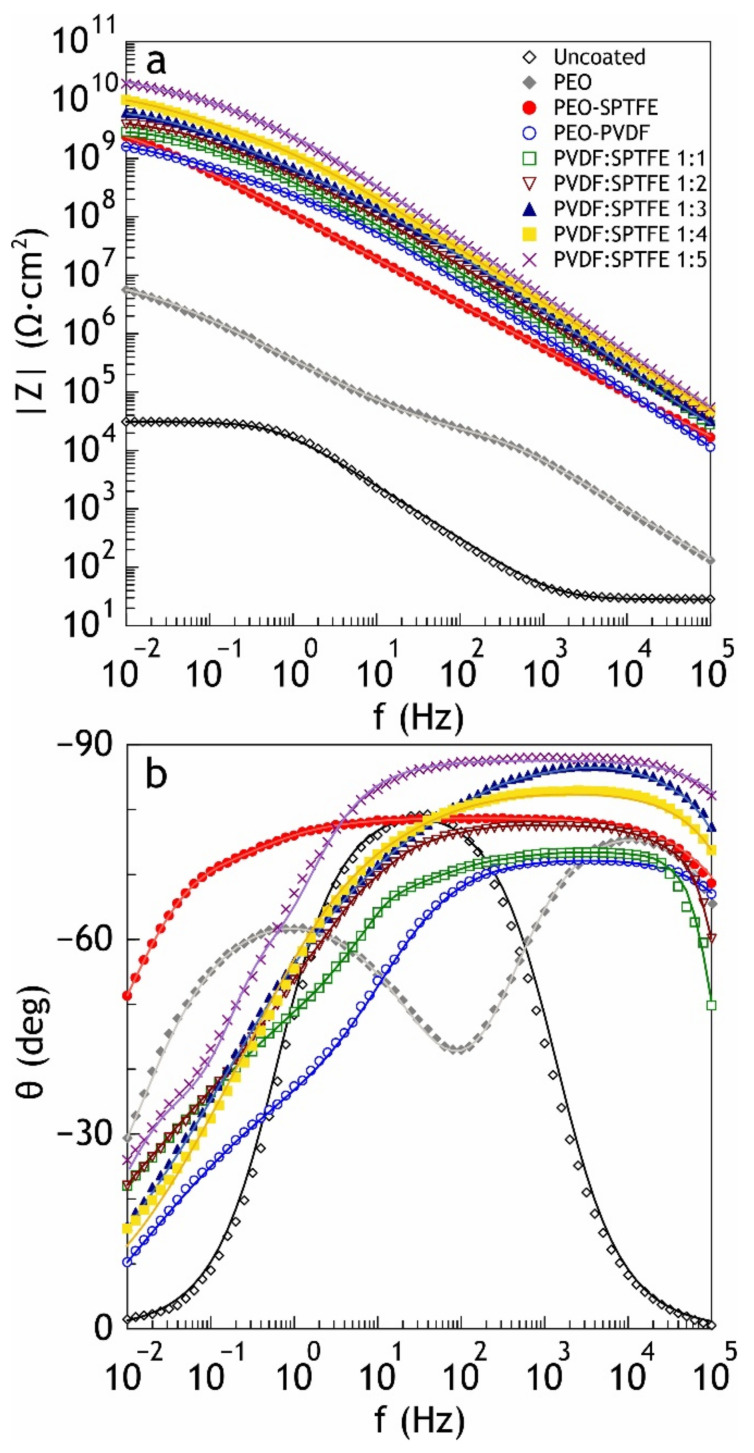
Bode plot (the impedance modulus |Z| (**a**) and the phase angle theta on the frequency (**b**) dependencies) obtained in 3 wt.% NaCl for the bare AMg3 aluminum alloy and the coated samples.

The potentiodynamic polarization curves of the bare aluminum alloy and the coated samples are shown in Figure 5. The calculated values of electrochemical parameters are presented in Table 2. In the examined range of potentials, the curve for the bare aluminum alloy has a form that is characteristic for this metal; after the cathodic part of the curve, a breakdown of the natural oxide/hydroxide film occurs with a corresponding sharp increase in the values of current density with the development of the corrosive process. Polarization curves obtained for the coated samples are located in the zone of substantially lower currents as compared to the curve for bare alloy and exhibit significant inhibition of both anodic and cathodic reactions. Figure 6 shows the results of impedance measurements in the form of Bode plots (the impedance modulus |Z| and the phase angle theta on the frequency dependencies). The values of the phase angle versus frequency dependence for the uncoated sample at middle and low ranges are due to the presence of a thin film of natural oxide on the sample surface and characterize the material as requiring protection. The spectra for the samples with the composite coatings confirm high barrier properties. The values of the phase angle *Θ* of the coated samples exhibit capacitive behavior and have a tendency to decrease to −90° at high and middle frequencies with an increasing SPTFE ratio.

As a result of the evaluation of electrochemical characteristics of the samples, it was revealed that formed protective layers significantly decrease the corrosion current density *Ic* of the samples. Thus, for the samples with the combined composite coating, *Ic* is 7.5 × 10^−12^ A·cm^−2^, which is more than five orders of magnitude lower than this parameter for an uncoated aluminum (*Ic* = 1.1 × 10^−6^ A·cm^−2^). *Ic* for CC-SPTFE is 8.1 × 10^−11^ A·cm^−2^, and a CC-PVDF layer is characterized by *Ic* = to 6.2 × 10^−11^ A·cm^−2^, which is more than four orders of magnitude lower than *Ic* for an uncoated aluminum. The difference in values of corrosion current density obtained from PDP curves and local current density determined using SVET is the result of different experimental conditions (different ratio of the studied surface to the volume of aggressive medium and different time of exposure of specimen to solution). Moreover, SVET is a very precise technique that provides the local values of the current density evaluated on the microscale, whereas PDP gives a value of the corrosion current density for the whole sample surface exposed to the electrolyte.

**Figure 7:**
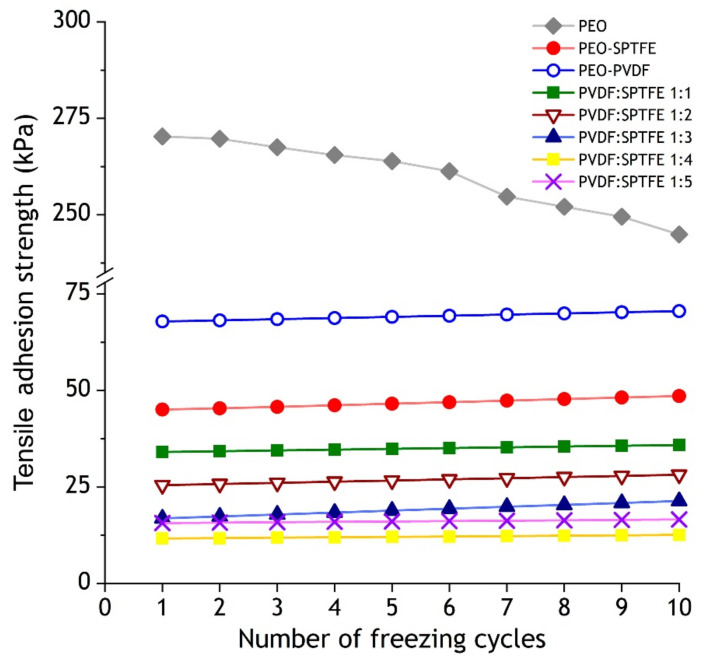
Dependence of adhesion strength on number of freezing cycles.

The ice pull adhesion strength (Figure 7) for the PEO coating is 270 kPa. The application of a PVDF layer leads to a fourfold decrease in the adhesion strength of ice (68 kPa). The treatment with SPTFE reduces the adhesion strength by six times (45 kPa). The maximum reduction in ice adhesion equal to 22.1 times (12.2 kPa) is provided by a superhydrophobic combined coating. After 10 tests, the value of the adhesive strength for the PEO coating reduces to 245 kPa, which most likely indicates the partial detachment of the coating’s weakly bound texturing elements forming its surface relief. However, all of the polymer-containing coatings demonstrate stable values.

## 4. Conclusions

As a result of the presented study, the methods of formation of fluorine-containing composite coatings with icephobic properties were suggested.

The results of analysis of the conducted studies revealed the following:

- The dip-coating method of formation of PVDF-containing layers allowed us to increase the uniformity of PEO coating surface relief, electrochemical properties and wettability. However, due to the absence of surface relief multimodality, the contact angle for these coatings attained 100.5° ± 1.8°, which did not allow us to classify the coating as superhydrophobic. Despite this, the formation of CC-PVDF coatings led to a fourfold decrease in ice pull adhesion strength (in comparison with the base PEO layer). This is probably explained by a significant reduction in surface roughness.
- The treatment of a PEO layer with SPTFE solution also resulted in a decrease in the number of pores and microdefects. The formation of a fluoropolymer layer led to advanced corrosion resistance and a decrease in wettability of the surface layer. The reduction in the pull ice adhesion strength of CC-SPTFE was 6 times, compared to the PEO-coating.
- The coatings obtained by combined PVDF–SPFTE treatment using a dip-coating method had the best wetting and corrosion resistance and icephobic properties. The pull ice adhesion strength of the combined coating was 22.1 times greater than that of the PEO layer.
- The data of SVET/SIET local scanning measurements indicated high protective properties of the CC-PVDF, CC-SPTFE and PVDF–SPFTE composite coatings. During the local studies, the formation of any pittings or defects was not detected.

## Figures and Tables

**Table 1:** Wetting characteristics of the samples.

Sample	CA, °	RA, °
PEO	28.3 ± 1.2	–
PEO–SPTFE	130.5 ± 2.1	–
PEO–PVDF	100.5 ± 1.8	–
PVDF–SPTFE 1:1	110.7 ± 0.5	–
PVDF–SPTFE 1:2	116.3 ± 0.7	–
PVDF–SPTFE 1:3	132.1 ± 1.7	–
PVDF–SPTFE 1:4	160.5 ± 3.8	6.3 ± 0.7
PVDF–SPTFE 1:5	155.0 ± 1.5	8.3 ± 0.6

**Table 2:** Calculated electrochemical parameters of the samples.

Sample	*E*_c_ [V vs. Ag/AgCl]	*I*_c_ [A·cm^−2^]	*R_p_* [Ω·cm^2^]	*|Z|*_f = 0.01 Hz_ [Ω·cm^2^]
Uncoated	−0.67	1.1 × 10^−6^	2.4 × 10^4^	2.9 × 10^4^
PEO	−0.87	8.4 × 10^−8^	1.9 × 10^5^	5.6 × 10^6^
PEO–SPTFE	−0.87	6.2 × 10^−11^	3.8 × 10^9^	2.2 × 10^9^
PEO–PVDF	−0.82	8.1 × 10^−11^	3.2 × 10^9^	1.9 × 10^9^
PVDF–SPTFE 1:1	−0.53	7.2 × 10^−11^	2.7 × 10^9^	3.0 × 10^9^
PVDF–SPTFE 1:2	−0.57	5.6 × 10^−11^	8.8 × 10^9^	3.9 × 10^9^
PVDF–SPTFE 1:3	−0.57	1.5 × 10^−11^	1.7 × 10^10^	5.9 × 10^9^
PVDF–SPTFE 1:4	−0.57	8.9 × 10^−12^	3.7 × 10^10^	1.1 × 10^10^
PVDF–SPTFE 1:5	−0.59	7.5 × 10^−12^	3.9 × 10^10^	2.0 × 10^10^

## Data Availability

Data presented in this study are available on request from the corresponding author.
